# IUC26696-94 Enfortumab Vedotin Plus Pembrolizumab Outcomes in Advanced Urothelial Cancer: Real-World Data

**DOI:** 10.1093/oncolo/oyag286.003

**Published:** 2026-08-21

**Authors:** S Peroos, A Saeed, S Jenkinson, A Clifford, M Prentice, S Mansukhani

**Affiliations:** Royal Free London NHS Foundation Trust - United Kingdom; Royal Free London NHS Foundation Trust - United Kingdom; Royal Free London NHS Foundation Trust - United Kingdom; Royal Free London NHS Foundation Trust - United Kingdom; Royal Free London NHS Foundation Trust - United Kingdom; Royal Free London NHS Foundation Trust - United Kingdom

## Abstract

**Background:**

Enfortumab vedotin plus pembrolizumab (EV-P) is the new standard first-line treatment with EV302/KeynoteA39 demonstrating OS, PFS and ORR benefit for locally advanced or metastatic urothelial carcinoma (la/mUC) compared with platinum-based regimes. We analysed real-world data to review the impact of EV-P on patient age-stratified outcomes.

**Methods:**

Retrospective analysis was performed on patients with histologically confirmed la/mUC treated with EV-P at Royal Free London Trust (RFL) between June 2025 and June 2026. Baseline demographic, radiological response and toxicity data were analysed.

**Results:**

29 patients commenced treatment in the 12-month period. Median age was 75 years (IQR: 69.6-80.2), and 62% were male. Upper tract urothelial carcinoma (UTUC) accounted for 66% of cases. 93% had metastatic vs 7% locally advanced disease. The longest duration of treatment was 11 cycles. ORR was 75.0% (15/20; 95% CI 53.3–88.8%), including 4 complete responses (CR) (20.0%; 95% CI 50.9–91.3%) and 11 partial responses (55.0%; 95% CI 31.5–76.9%). Stable disease occurred in 2 patients (10.0%; 95% CI 1.2–31.7%) and progressive disease in 3 (15.0%; 95% CI 3.2–37.9%). 2 patients with CR or near CR were referred for cytoreductive surgery in view of treatment-related toxicities necessitating discontinuation. At the data cut-off, 4 patients died. Adverse effects are summarised in Table 1.

**Conclusions:**

In a real-world cohort characterised by older age, median 75 vs 69 in EV-302, greater comorbidity and a higher proportion of UTUC, EV-P achieved an ORR of 75% (vs 67.7% in EV-302). These early findings support the effectiveness and tolerability of EV-P in clinical practice, with patients becoming candidates for consolidative therapy for local control. This is especially important in UTUC patients, where there is currently limited evidence for neoadjuvant therapy.

**IUC26696-94 Table 1:** Summary of Toxicities

CTCAE Grade	1	2	3	4
**Enfortumab Vedotin**				
**Fatigue**	46.6%	3.3%	3.3%	0
**Altered appetite**	13.3%	0	0	0
**Peripheral neuropathy**	30%	6.7%	0	0
**Skin**	23.3%	0	0	0
**Serious infection**	0	0	13.3%	3.3%
**Pembrolizumab**				
**Skin**	23.3%	0	6.7%	0
**Colitis**	3.3%	6.7%	3.3%	0
**Endocrinopathy**	6.7%	0	0	0
**Myocarditis**	0	0	3.3%	0
**Neurotoxicity**	0	3.3%	0	0

